# A placebo-controlled trial of folic acid and betaine in identical twins with Angelman syndrome

**DOI:** 10.1186/s13023-019-1216-0

**Published:** 2019-10-22

**Authors:** Julia Han, Terry Jo Bichell, Stephanie Golden, Irina Anselm, Susan Waisbren, Carlos A. Bacino, Sarika U. Peters, Lynne M. Bird, Virginia Kimonis

**Affiliations:** 1 Division of Genetics and Genomic Medicine, Department of Pediatrics, University of California at Irvine, 101 The City Drive South, Orange, CA 92868 USA; 2 Consortium for Outcome Measures and Biomarkers for Neurodevelopmental Disorders, Nashville, TN USA; 3 Division of Genetics and Metabolism, Department of Medicine, Boston Children’s Hospital and Harvard Medical School, Boston, MA USA; 4 Department of Neurology, Boston Children’s Hospital and Harvard Medical School, Boston, MA USA; 5 Department of Molecular and Human Genetics, Baylor College of Medicine, Houston, TX USA; 6 Department of Pediatrics, Vanderbilt University, Vanderbilt Kennedy Center for Research on Human Development, Nashville, TN USA; 7 Department of Pediatrics, University of California, San Diego, CA USA; 8 Division of Genetics/Dysmorphology, Rady Children’s Hospital San Diego, San Diego, CA USA

**Keywords:** Angelman syndrome, Deletion, Methylation, Silencing, Methyl donors, Betaine, Folic acid

## Abstract

**Background:**

Angelman syndrome (AS) is a neurodevelopmental disorder that is caused by maternal genetic deficiency of a gene that encodes E6-AP ubiquitin-protein ligase (gene symbol *UBE3A*) mapping to chromosome 15q11-q13. AS leads to stiff and jerky gait, excess laughter, seizures, and severe intellectual disability. In some parts of the brain, the paternally inherited *UBE3A* gene is subject to genomic imprinting by the action of the *UBE3A*-antisense transcript (*UBE3A-ATS*) on the paternally inherited allele. Consequently, only the maternally inherited *UBE3A* gene is expressed in mature neurons. AS occurs due to deletions of the maternal 15q11 − 13 region, paternal uniparental disomy (UPD), imprinting center defects, mutations in the maternal *UBE3A* gene, or other unknown genetic malfunctions that result in a silenced maternal *UBE3A* gene in the specific imprinted regions of the brain.

**Results:**

A potential treatment strategy for AS is to increase methylation of *UBE3A-ATS* to promote expression of the paternal *UBE3A* gene and thus ameliorate the clinical phenotypes of AS. We treated two sets of male identical twins with class I deletions with a 1 year treatment trial of either betaine and folic acid versus placebo. We found no statistically significant changes in the clinical parameters tested at the end of the 1 year trial, nor did we find any significant adverse events.

**Conclusions:**

This study tested the hypothesis that by increasing the methylation of the *UBE3A-*antisense transcript in Angelman syndrome to promote expression of the silenced paternal *UBE3A* gene we may ameliorate the clinical phenotypes of AS. We treated two sets of identical twins with placebo versus betaine and folic acid. Although this study represented a novel approach to treating Angelman syndrome, the differences in the developmental testing results was not significant. This paper also discusses the value of monozygotic twin studies in minimizing confounding variables and its utility in conducting small treatment studies.

**Trial registration:**

NCT00348933. Registered 6 July 2006.

## Introduction

Angelman syndrome (AS), first described by Harry Angelman in 1965, is a neurodevelopmental disorder that is characterized by severe congenital intellectual disabilities, ataxia, unusual facial appearance, and a happy/sociable disposition) [1]. AS is caused by de novo maternal deletions of chromosome 15q11-q13 (70–80%), intragenic mutations in the maternally inherited UBE3A within chromosome15q11-q13 (10–20%), paternal uniparental disomy (UPD) for chromosome 15q11-q13 (3–5%), or imprinting defects within chromosome 15q11-q13 that alter the expression of maternally inherited UBE3A (3–5%) [2, 3]. *UBE3A* is expressed from both the paternal and maternal chromosome in somatic tissues and in many parts of the brain; however, only the maternally inherited gene is expressed in mature neurons [4]. The *UBE3A* antisense transcript (*UBE3A*-ATS) is presumed to be responsible for silencing the paternal *UBE3A* expression in these cells. It is hypothesized that increased methylation of *UBE3A*-ATS may increase expression of the paternally inherited *UBE3A* gene and ameliorate the phenotypes in Angelman syndrome [5]. The use of methyl donors in the diet were hypothesized to promote such an effect. Our treatment rationale was to increase global methylation levels in the brain to promote gene expression from the silent *UBE3A* paternal allele through a one-year treatment trial of betaine and folic acid [6, 7]. This case report limits biological and environmental variability by studying a small sample of two sets of identical twins. In a larger study, confounding variables may affect the results.

## Methods

### Experimental subjects

Two sets of identical twin male siblings were studied under an Institutional Review Board-approved protocol at Boston Children’s Hospital (Committee on Clinical Investigation) (twins A and B) and Rady Children’s Hospital, San Diego (UC San Diego Pediatric Institutional Review Board) (twins C and D). Written informed consent was obtained from parents before enrollment. Recruitment followed the standards of the Declaration of Helsinki. Inclusion criteria for the study included laboratory confirmation of diagnosis for common deletion, UPD, imprinting defect, or UBE3A mutation. Patients with a clinical diagnosis of AS but no identifiable molecular genetic abnormality were not be eligible to enroll. Patients who were not clinically stable were excluded from the study. All racial/ethnic were eligible for the study. All patients had Fluorescent in situ hybridization (FISH) studies which showed deletions on chromosome 15, and DNA methylation studies were diagnostic of AS. Additional studies performed by MLPA to determine deletion size indicated that all subjects had a larger class 1 deletion.

### Clinical protocol

The two sets of identical twins were studied in a placebo-controlled double-blind treatment trial of betaine and folic acid treatment. Twins A, C and D were randomized independently using a centralized method so their assignment was random. Twin A was assigned placebo randomly and twin B was assigned betaine and folic acid. Twin C was assigned placebo and twin D was assigned betaine and folic acid. Twins A and B were enrolled at 8 years and 5 months respectively, twin C at 5 years and 8 months and twin D when 7 years old. All twins completed the study.

### Evaluation

At baseline, 6, and 12 months the patients underwent clinical evaluations from a clinical geneticist, neurologist, and psychologist, developmental and laboratory evaluations, and every 3 months the parents were asked to complete a questionnaire. The parent questionnaire monitored the child’s overall behavior, i.e. feeding difficulties, oral sensitivity, sleep, drooling, toilet training, tremoring sitting, standing, nonverbal communication, hypermotoric behavior, and attention span.

The developmental evaluations included a total of four tests. Bayley Scales of Infant Development, Second Edition (BSID-II) [8] and the Vineland Adaptive Behavior Scales – Interview Edition [9] were used to assess cognitive and motor skills. The Preschool Language Scale, Third Edition (PLS-III) [10] and the MacArthur Developmental Inventory [11] were used to assess communication skills. ADOS and ADI testing was not performed in one set of twins only. Laboratory testing included CBC, urinalysis, folate levels at Quest Diagnostics in Cambridge, MA, and plasma betaine and amino acid levels at Baylor College of Medicine.

## Case report

### Twins A and B

Twin A and B were idenitical monoamniotic twins born to a 24-year-old G3 P2 mother and a 31-year-old father. The pregnancy was complicated by twin gestation diagnosed at 7 weeks, and gestational diabetes in the last month of pregnancy, which was controlled by diet. Delivery by repeat Caesarean section at 35 ½ weeks of secondary to prior uterine surgery for an ectopic pregnancy.

#### Twin A

The neonatal period was unremarkable. At two and a half months he had an incarcerated left inguinal hernia that was repaired surgically. He has had a history of clinical seizures since the age of 15 months controlled by medication including topiramate, clonazepam, and sodium valproate.

On examination at 8.4 years his height was 127.3 cm (50%), weight 25.9 kg (50%), and head circumference 52.5 cm (50%). Physical findings included brachycephaly, mid-facial retrusion, prognathism, macrostomia, wide-spaced teeth, tongue thrusting and strabismus. General examination of heart, lungs, and abdomen was unremarkable. On neurological exam, cranial nerves were grossly intact. His motor examination was notable for truncal hypotonia and increased tone in all extremities. He had jerky movements and a wide-based gait with his arms held in flexion when walking. His EEG at age 8 years was abnormal with widespread delta slowing maximal posteriorly at the fully alert state but dominant frontally in addition to delta, notched delta blunted spike waves or frank spike waves were seen of which one was associated with a clinical event (grunting).

#### Twin B

Twin B had mild respiratory distress at birth and was admitted to the Neonatal Intensive Care Unit for 10 days for antibiotic therapy for pneumonia. He began to have seizures at 15 months of age, during which he would tighten his jaw, make growling noises, and have rhythmic tremors. His seizures were controlled by medication EEG obtained at 8 years was suggestive of an active seizure disorder, very characteristic of Angelman syndrome with widespread posterior discharges consisting of delta, notched delta and spike waves. On examination at 8 y. and 5 mo., his height was 128.2 cm (50%), weight 26 kg (50%) and head circumference 53 cm (50-75%). Craniofacial features were identical to his twin. Unlike his twin, he aexhibited mild scoliosis. Both twins were diagnosed with Angelman syndrome at 16 months of age through FISH testing with probes from D15S11 and GABRB3 that showed a typical Angelman syndrome Class I deletion at chromosome 15. DNA methylation studies were conducted and concluded to be diagnostic of AS.

### Twins C and D

Twins C and D were born to a 29 year old G2 P0 mother and 34 year old father. The pregnancy was naturally conceived and a routine ultrasound at 16 weeks gestation revealed monoamniotic monochorionic twins. There were no exposures during the pregnancy. An ultrasound disclosed no problems. The pregnancy was complicated by preterm contractions beginning at 29 weeks, treated with bedrest and terbutaline, and HELLP syndrome, treated with MgSO_4_. At 34 weeks gestation, rupture of membranes occurred, and induction of labor was undertaken. Twin D was born vaginally, but twin C was delivered by emergency caesarean section due to cord prolapse.

Both twins had severe muscle hypotonia that persisted beyond a year of age and were diagnosed with Prader-Willi syndrome based on FISH at 13 months. Breast feeding was not successful due to a weak suck and they were fed breast milk in a bottle with a preemie nipple for 1 month and then transitioned to formula. Their coloring was lighter than expected for the family. At 2 years of age, the diagnosis was changed to Angelman syndrome based on DNA methylation results. Twin C was examined at 5 years and 8 months and twin D was examined at 7 years of age. Their chromosome 15q11-q13 deletion associated with Angelman syndrome was characterized as a Class I deletion.

#### Twin C

Twin C weighed 2.15 kg, measured 47 cm, and had Apgar scores of 4 and 7. He stayed for 9 days in the Neonatal Intensive Care Unit for temperature regulation and feeding issues. He had febrile seizures in association with a respiratory infection at age 4 months, and non-febrile convulsions began at age 28 months. He had several episodes of status epilepticus and failed management with 6 anti-epileptic medications. Severe failure to thrive necessitated percutaneous gastrostomy tube placement at 3 years of age. When evaluated at age 5 years 8 months, he weighed 16 kg (12%), measured 112 cm (70%), and had a head circumference of 49 cm (75%). He had microbrachycephaly with an occipital ridge, exotropia, midface retrusion, prognathism, and macrostomia with widely spaced teeth. He had a moderate rightward thoracic and leftward lumbar scoliosis and pronated ankles. He had generalized muscle hypotonia.

#### Twin D

Twin D weighed 2.13 kg, measured 47 cm, and had Apgar scores of 4 and 7. He was gavage fed for 3 days and discharged home at 9 days. Exotropia and sleep disturbance were noted from infancy. Food refusal and severe failure to thrive necessitated percutaneous gastrostomy tube placement at 3 years of age. There were multiple episodes of otitis media and pneumonia, the latter presumed due to aspiration. Gagging and gastroesophageal reflux disease resulted in many episodes of emesis per day.

Seizures with fever began at 4 months, and afebrile seizures started at 28 months of age. Nearly all developmental skills were lost until seizure control was achieved approximately several months later, following which skills were regained slowly. On examination at 7 years of age, he weighed 19.5 kg (10%), measured 119.3 cm (30%) and had a head circumference of 49.4 cm (50%). He had alternating exotropia, midface retrusion, prognathism, widely spaced teeth, and mild rightward thoracic and leftward lumbar scoliosis. His truncal tone was low, and there was high extensor tone in all extremities.

## Developmental testing and results

Developmental testing was performed by psychologists at Boston Children’s Hospital (twins A and B) and Rady Children’s Hospital San Diego (twins C and D). Table 1 summarizes features of all four twins. Table 2 presents raw scores at baseline, 6 months and 12 months after study enrollment (treatment or placebo). For the most part, raw scores are reported, since performance was far below the designated basal score in the norm tables of the manuals. We also present the standard score for the Vineland Adaptive Behavior Composite, which has a normative mean of 100 ± 15.

**Table 1:** Summary of clinical features in twin pairs

Twin	Gestation age	Birthweight	Apgar Scores (1,5 min)	Deletion Size	ADOS	Seizure Onset	Medications
A	35.5 weeks	2.75 kg	8, 9	Class 1	Not tested	15 months	Topamax, Clonazepam, Valproate
B	35.5 weeks	2.47 kg	3, 6	Class 1	Not tested	15 months	Topamax, Clonazepam, Valproate
C	34 weeks	2.15 kg	4, 7	Class 1	Autism	4 months	Miralax, Lamictal, Zonagran, Melatonin
D	34 weeks	2.13 kg	4, 7	Class 1	Autism	4 months	Miralax, Lamictal, Zonagran, Klonopin, Melatonin

**Table 2:** Developmental Testing Scores at baseline, 6 months and at 12 months

Variables (Raw Scores) at 0, 6 and 12 months	TWIN	0	6	12	^c^Score 6-0	^c^Score 12-6	^c^ Total Score
Bayley Mental	A^a^	76	83	90	7	7	14
	B^b^	77	78	78	1	0	1
	C^a^	70	77	78	7	1	8
	D^b^	73	73	76	0	3	3
Bayley Motor	A	66	67	74	1	7	8
	B	66	67	73	1	6	7
	C	38	41	44	2	3	5
	D	51	50	47	-1	−3	−4
Preschool Language Auditory Comprehension	A	8	9	9	1	0	1
	B	8	8	9	0	1	1
	C	5	7	6	2	−1	1
	D	5	5	7	0	2	2
Preschool Language Expressive Communication	A	6	9	9	3	0	3
	B	5	9	8	4	−1	3
	C	6	6	6	0	0	0
	D	4	3	3	−1	0	−1
Preschool Language Composite	A	14	18	18	4	0	4
	B	13	17	17	4	0	4
	C	11	13	12	2	−1	1
	D	9	8	10	−1	2	1
*Vineland Communication	A	24	24	26	0	2	2
	B	24	23	25	−1	2	1
	C	12	14	16	2	2	4
	D	14	15	14	1	−1	0
*Vineland Daily Living	A	31	31	35	0	4	4
	B	29	27	33	−2	6	4
	C	7	6	6	−1	0	−1
	D	8	9	8	1	− 1	0
*Vineland Socialization	A	29	33	33	4	0	4
	B	27	29	33	2	4	6
	C	25	30	30	5	0	5
	D	23	26	17	3	−9	−6
*Vineland Adaptive Behavior Composite	A	164	117	131	−47	14	−33
	B	80	105	127	25	22	47
	C	139	131	106	−8	−25	−33
	D	145	136	123	−9	−13	− 22
*Vineland Adaptive Behavior Composite (standard scores)	A	29	28	27	−1	−1	−2
	B	29	–	26	–	–	−3
	C	32	30	32	−2	2	0
	D	33	42	28	9	−14	−5
MacArthur Communicative Developmental Inventory Phrases understood	A	25	25	16	0	−9	−9
	B	19	22	15	3	−7	−4
	C	2	0	0	−2	0	−2
	D	0	0	2	0	2	2
MacArthur Communicative Developmental Inventory Vocabulary comprehension	A	1	3	0	2	−3	−1
	B	1	2	0	1	−2	−1
	C	1	0	5	− 1	5	4
	D	3	2	8	−1	6	5
MacArthur Communicative Developmental Inventory production	A	0	0	1	0	1	1
	B	0	2	0	2	−2	0
	C	1	0	1	−1	1	0
	D	0	0	0	0	0	0

* Vineland did not include the motor skills portion because the twins were over age 6 years

The labels ^a^ for placebo, and the label ^b^ for drug (betaine and folic acid) treatment applies to all the testing groups. ^c^ refers to differences in the scores between baseline, 6 months and 12 months

*P*-values calculated through t-test were not statistically significant

**Fig. 1:**
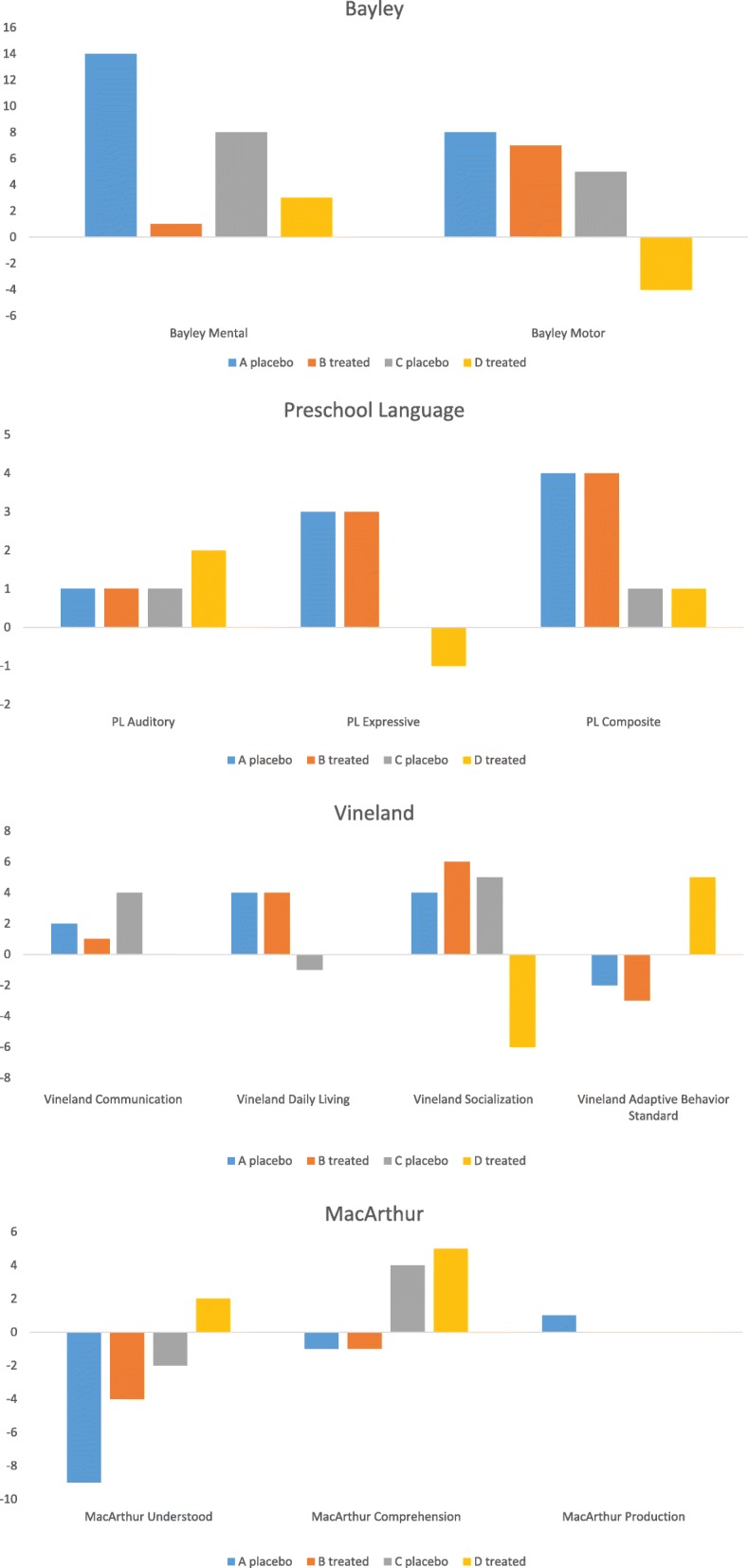
Graphs of total score differences in Bayley, Preschool Language, Vineland, and MacArthur tests across twin A, B, C, and D. *P-values calculated through Student’s t-test were not statistically significant (all *p*-values>0.05)

This case study did not demonstrate significant developmental differences between twin A (treated with placebo) and twin B (treated with betaine and folic acid) nor between twin C (treated with placebo) and twin D (treated with betaine and folic acid). There was no statistically significant difference between the two sets of twins. Table 2 delineates scores from all developmental testing over a 12-month period. Figure 1 summarizes the total score difference in each test for twins A, B, C, and D. All twins were below the normative mean (100 ± 15) of the Vineland Adaptive Behavior Composite, perhaps attributed to their large deletion classes (Table 2). Twins A and B exhibited higher Bayley Motor, Vineland Communication, Vineland Daily Living, and MacArthur Communicative Developmental Inventory Phrases Understood scores than twins C and D over a 12-month period. All twins exhibited similar scores with no significant improvement in Bayley Mental, Preschool Language Auditory Comprehension, Preschool Language Expressive Communication, Preschool Language Composite, Vineland Socialization, and Vineland Adaptive Behavior Composite. However, between the two sets of twins, twins C and D improved in MacArthur Communicative Developmental Inventory Vocabulary Comprehension while twins A and B worsened over a 12-month period (Fig. 1). There was no significant differences in the serum levels of betaine in the two groups.

## Discussion

Angelman syndrome is a neurodevelopmental disorder with severe mental impairments, neurological impairments, neurological defects, seizures, sleep disturbances, and lack of sleep among its major clinical manifestations [12, 13]. In hopes of mitigating these phenotypic characteristics, we treated a set of identical twins with either placebo or folic acid and betaine to increase methylation of *UBE3A*-ATS and hopefully increase expression of the paternally inherited UBE3A protein. Betaine is known to cross the blood brain barrier and animal studies also indicate that hippocampal slices actively accumulate betaine in a time, dose and osmolality dependent manner, resulting in peak intracellular concentrations four times extracellular concentrations within 8 h. Betaine thus has the potential to modulate neuronal excitability and induce the hippocampus to mediate learning and memory and promote cognition [14].

No consistent pattern of improvement however was seen in twins B or D, who received treatment, in comparison to twins A and C, who did not. These findings complement the studies of Peters et al. [7], in which betaine and folic acid treatment in 48 AS patients showed no statistically significant changes in the clinical phenotype. They found that children who were on medications and did not have a diagnosis of co-morbid autism showed an upward trend in their development; however, differences were not statistically significant [7]. The four patients in this case study were included in the larger study of Peters et al. [7]; however, we provide more details on the two sets of identical twins because of the power of monozygotic twin analysis.

Though the treatment had no statistical effect on any of these subjects via student’s t-test (all *p*-values> 0.05), these two sets of twins with Angelman syndrome are especially informative because both sets are matched for similarity for the larger deletion size (Class I), including scores and phenotype between twins and between sets of twins. The outcome measures used in this trial were designed for the general population and may not be appropriate for picking up incremental developmental changes in people with Angelman syndrome, as scores were almost at the floor of each of the developmental assays. However, scores for all four subjects on the developmental tests described here were remarkably similar and stable over time. All four of these subjects scored comparatively low compared to the remainder of the subject population, which may be due to their larger deletion size, or may be due to other variables related to multiple birth, such as prematurity. Previous studies have shown that patients with Class I deletion are at higher risk of autism than others with Angelman syndrome, and those subjects in the trial without autism showed a trend toward a beneficial effect of the treatment [7]. Twins C and D tested positive for autism via the Autism Diagnostic Observation Schedule [15]. Unfortunately, twins A and B were not tested for autism but clinically did not exhibit features of autism.

Monozygotic (MZ) twin studies in disease and epigenetic studies are valuable because there is a significant reduction of confounding variables and a smaller cohort that can provide useful data. MZ twin studies control for the effects of age, sex and genetic factors, which facilitates the assessment of treatment effects [16]. This study design has been used by many other researchers in the past. Oberfield [17] treated one set of identical twins diagnosed with nephropathic cystinosis with ascorbic acid versus placebo. Hunter [18] conducted a longitudinal, placebo-controlled, double blind trial of vitamin D supplements on identical female twins and concluded that they did not find any significant prevention of osteoporosis. Ferraresi [19] employed a placebo-controlled Light-Emitting Diode Therapy on one pair of identical twins and found significantly decreased levels of interleukin 1β (markers of inflammation) and myostatin (markers of muscle atrophy) in the treated twin. Ring [20] also treated one set of identical twins suffering from severe atopic eczema with grass pollen and saline versus placebo and found statistically significant improvement in clinical phenotype in the treated twin. In these MZ twin studies, the incorporation of MZ twins as identical controls led to clear and dependable data on the effect of novel treatments on very small sets of patients. Similarly in our study, data from the MZ twins minimized confounding variables, such as age, gender, cultural, environmental, or genetic factors, and allowed for reliable conclusions. We however did not find significant improvements with the betaine and folic acid treatment, nor did we find negative effects of the treatment in the treated twins.

## Conclusions

Betaine and folic acid treatment was shown to have no statistically significant effect on treated twins compared to control. The nature of this study as a monozygotic twin investigation adds validity to the controllability and consistency of the results, as many confounding factors have been accounted for.

## Data Availability

Tables and figures relevant to this case study are embedded in the manuscript.
